# An Invasive Vector of Zoonotic Disease Sustained by Anthropogenic Resources: The Raccoon Dog in Northern Europe

**DOI:** 10.1371/journal.pone.0096358

**Published:** 2014-05-22

**Authors:** Karmen Süld, Harri Valdmann, Leidi Laurimaa, Egle Soe, John Davison, Urmas Saarma

**Affiliations:** Department of Zoology, Institute of Ecology and Earth Sciences, University of Tartu, Tartu, Estonia; Kent State University, United States of America

## Abstract

The raccoon dog (*Nyctereutes procyonoides*) is an introduced species in Europe with a continually expanding range. Since the species is capable of affecting local ecosystems and is a vector for a number of severe zoonotic diseases, it is important to understand its food habits. Raccoon dog diet was studied in Estonia by examining the contents of 223 stomach samples collected during the coldest period of the year, August to March, in 2010–2012. The most frequently consumed food categories were anthropogenic plants (e.g. cereals, fruits; FO = 56.1%) and carrion (e.g. carcasses of artiodactyls and carnivores; FO = 48.4%). Carrion was also the only food category that was consumed significantly more frequently by raccoon dogs exhibiting symptoms of sarcoptic mange than by uninfected animals. Small mammals, which represent intermediate hosts for the zoonotic tapeworm *Echinococcus multilocularis*, were more commonly recorded in samples also containing anthropogenic plants than expected by chance. Comparison of raccoon dog and red fox (*Vulpes vulpes*) diet in Estonia revealed higher overlap than found elsewhere in Europe, with ‘carrion’ and ‘anthropogenic plants’ making up the bulk of both species’ diet; however, raccoon dogs were more omnivorous than red foxes. Our results suggest that while the use of most food categories reflects the phenology of natural food sources, ‘anthropogenic plants’ and ‘carrion’ provide an essential resource for raccoon dogs during the coldest period of the year, with the latter resource especially important for individuals infected with sarcoptic mange. Since both of these food categories and small mammals are often found at supplementary feeding sites for wild boar (*Sus scrofa*), this game management practice may facilitate high densities of mesocarnivores and promote the spread of some severe zoonotic diseases, including alveolar echinococcosis, trichinellosis, rabies and sarcoptic mange.

## Introduction

Introduced terrestrial vertebrates are often species with generalist feeding habits and short generation times, allowing them to adapt to different environments and cause a range of ecological and economic problems [1]. Genovesi *et al*. [2] reported 117 alien mammal species in Europe, of which at least 58 are known to cause environmental or health problems, and 27 have been found to directly affect native species. One of the most successful invasive carnivores in Europe is the raccoon dog (*Nyctereutes procyonoides*) [3], which is suspected of causing damage to native fauna through its feeding habits. Although insects, plants and small mammals are the main food objects for raccoon dogs in Europe [4], the species is an opportunistic feeder and is considered a potential threat to ground nesting birds and amphibians [5], [6], [7], [8].

The raccoon dog is also an important vector of zoonotic disease [3]. In Estonia it has been identified as a vector for rabies [9], trichinellosis [10], sarcoptic mange and alveolar echinococcosis. Rabies has been eradicated from Estonia following a successful anti-rabies vaccination campaign, initiated in 2005 [11]. However, in recent years not only has the raccoon dog population started to increase, but so has the number of animals infected with sarcoptic mange, a highly contagious zoonosis caused by the burrowing mite *Sarcoptes scabiei*. The parasite infects a wide variety of wild and domesticated mammals, including humans [12]. Sarcoptic mange is known to impose considerable nutritional stress on individual animals [13] and ultimately severely reduce population densities of wild canids, especially red foxes and raccoon dogs [3].

Recently, the raccoon dog has been identified as an important host species for the tapeworm *Echinococcus multilocularis*, causative agent for the severe infectious disease, alveolar echinococcosis, which can be fatal for humans [14]. In Estonia, this parasite has been found in red foxes [15] and recently also in raccoon dogs (Laurimaa *et al*., unpublished). The principal mode of transmission for *E. multilocularis* involves an intermediate mammal host, usually in the form of a small mammal. Since earlier studies have indicated that small mammals and carrion represent potentially important food items for raccoon dogs in Estonia [5], [16], detailed studies of the species’ feeding habits are essential to evaluate its potential to transmit alveolar echinococcosis and other zoonotic diseases.

Since the introduction of raccoon dogs to Estonia in 1950 [17] three studies of the species’ diet have been carried out. According to the two earliest studies, by Naaber [5] and Laanetu [6], small mammals and plants formed the bulk of raccoon dog diet. However, both studies targeted relatively small study areas. The most recent study, by Rätsepp [16], suggested that ungulate carrion and anthropogenic plants (e.g. cereal crops and fruit) are important food sources, but unfortunately the sample size was too small to draw firm conclusions. Wildlife management has changed considerably in Estonia during recent decades, and it is reasonable to assume that food resources and therefore also the feeding habits of raccoon dogs have also changed. One potentially significant factor influencing raccoon dog diet in recent years is the increased number of artificial feeding sites created for wild boar (*Sus scrofa*), which provide animals with greater access to anthropogenic plants and carrion.

We examined raccoon dog stomach contents collected throughout Estonia to establish an overview of the species’ current feeding habits and dietary differences between the sexes and in relation to infection with sarcoptic mange. We also compared the diet of the raccoon dog with that of the red fox (*Vulpes vulpes*), another ecologically important mesopredator and vector for zoonotic disease. We hypothesize that anthropogenic food items have become an important source of supplementary food for raccoon dogs, and may be especially critical during winter, when natural food is in short supply and conditions are energy demanding (in Estonia, raccoon dogs spend part of the winter in hibernation, but emerge relatively frequently to search for food). Moreover, as ungulate numbers and hunting bags have increased considerably since the 1960s, we predict that carrion may now play an important role in raccoon dog diet. We also hypothesize that there is a difference in the feeding habits of individuals infected with sarcoptic mange, compared to non-infected individuals: due to significant energy loss, infected animals might prefer energy-rich and easily accessible food items.

## Materials and Methods

Carcasses of 252 raccoon dogs were collected in Estonia between August and March, 2010–2012 (Figure S1; Table S1). Of these, 249 samples were collected from animals legally harvested by hunters for purposes other than this project, while three were from animals killed in traffic accidents. After weighing and sexing, stomachs were removed and stored at −80°C for at least five days before proceeding with further analyses, in order to avoid the risk of *Echinococcus* tapeworm infection [18], since both *E. multilocularis* [15] and *E. granulosus* [19] have recently been found in Estonia.

For analysis, stomachs were opened and the contents washed through a sieve (1 mm mesh size). To quantify diet composition and calculate food niche breadth, we measured the volume (ml) of each component by water displacement [20]. In total, 223 stomach contents contained some food items, while 26 were empty.

Identifiable material was sorted into nine food categories: 1) ‘small mammals’ (Rodentia, Insectivora), 2) ‘carrion’ (Cervidae, Suidae, Carnivora), 3) ‘birds’, 4) ‘amphibians’, 5) ‘fish’, 6) ‘invertebrates’, 7) ‘anthropogenic plants’ (cereals, fruits), 8) ‘natural plants’ (wild berries, grass, etc.), 9) ‘garbage’ (remains of vegetables, sausage skins, etc.). Mammal and bird remains were identified to the lowest taxon possible. For identification of mammals we analysed hair cuticular scale pattern, medulla and cross-section according to Teerink [21] and a hair reference collection. Teeth were identified according to Kaal [22]. Reference materials were also used for identification of bird feathers. It is important to consider the possibility that conspecific hair in diet studies could be due to grooming [23]. With this in mind, we included raccoon dog hairs as a dietary component only when the volume of hairs in a stomach was >20 ml, which we consider unlikely to result from grooming. Moreover, the time of intensive moulting does not coincide with our study period [24].

We calculated the frequency of occurrence of each food type (FO = number of stomachs containing a specific food item/total number of stomachs). To calculate food niche breadth we used Levińs index which is based on volumes of food items [25]. To examine seasonal differences in diet composition the data were grouped into two temporal categories: 1) autumn (August–November), a period of intensive foraging and fat accumulation; and 2) winter (December–March), the period when raccoon dog activity is reduced due to cold weather. As the hunting time of four raccoon dogs was unknown, we excluded these individuals from the seasonal analysis. We could not obtain study material from the summer period since raccoon dogs are rarely hunted then in Estonia. Of all analysed stomach contents, 33 belonged to raccoon dogs exhibiting symptoms of sarcoptic mange. Differences in diet composition between sexes and seasons and in relation to sarcoptic mange infection were tested using PERMANOVA (with Bray-Curtis distance; function adonis in R package vegan) [26] and visualised using non-metric multidimensional scaling (function metaMDS in R package vegan) [26]. Individual food items that varied in relation to infection, sex or season were identified using 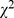-tests. To determine the potential impact of infected individuals on our results, detailed analysis of season and sex differences was carried out on two datasets: 1) ‘total’ (uninfected plus infected individuals, n = 223); and 2) only ‘uninfected’ individuals (n = 190).

To assess the co-occurrence of food categories in raccoon dog diet, we calculated the C-score [27] for all pairs of food types. To generate a distribution of C-scores that could be expected if food types were distributed randomly with respect to one another, we generated 999 random matrices with fixed row and column occurrences, and recalculated all pairwise C-scores for each matrix. Observed C-scores were standardised by subtracting the mean of the randomised C-scores and dividing by the standard deviation of the randomised values. The significance of effects was estimated from the number of randomised C-scores more extreme than the observed value and corrected using the false discovery rate approach [28]. Analyses were carried out on the full data set and separately for autumn and winter samples.

We also compared our data with the results of earlier diet studies performed in Estonia by Rätsepp [16] and Naaber [5] (see Figure S2; Information S1).

Raccoon dog diet from autumn and winter in Estonia was also compared with red fox diet (stomach contents, n = 92) from the same periods and areas (Soe *et al*., unpublished) using PERMANOVA. Dietary overlap between male and female raccoon dogs, and between raccoon dogs and red foxes was evaluated using Pianka’s index [29]. A Mann-Whitney U-test was used to test for differences in stomach volume between sexes. For these analyses, small mammals and carrion were considered as one food category ‘mammals’, as it was not possible to estimate the volume of hairs separately for these two categories.

## Results

### General Diet Composition

The most frequently consumed food items were ‘anthropogenic plants’ (FO = 56.1%) and ‘carrion’ (FO = 48.4%); ‘small mammals’, ‘invertebrates’ and ‘natural plants’ were recorded nearly two times less frequently (Table 1). ‘Anthropogenic plants’ included cereals (rye *Secale cereal*e, wheat *Triticum* spp., oat *Avena sativa*), whereas ‘carrion’ consisted mainly of artiodactyls (FO = 34.5%), such as wild boar (*Sus scrofa*) and roe deer (*Capreolus capreolus*), but also carnivores (FO = 17.5%). Among carnivores, the most frequently found remains were from raccoon dogs (FO = 9.0%) (Table 2). Among ‘small mammals’ (FO = 29.6%), voles (*Microtus* spp., and especially the bank vole *Myodes glareolus*) and shrews (*Sorex* spp.) were most frequently present (Tables 1 and 2). Of bird feathers, 37.9% were identified as Passeriformes, and 6.9% belonged to the hazel grouse (*Tetrastes bonasia*); the remaining feathers could not be identified due to severe degradation. Based on the nine food categories presented in Table 1, the food niche breadth was 2.83.

**Table 1:** Frequency of occurrence (FO) of different food items in raccoon dog stomachs in different seasons: autumn (August–November), winter (December–March).

Food category	Total	Autumn	Winter	Autumn/Winter			
		FO%		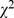	p-value	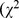	p-value)
**Small mammals**	30(29)	21(21)	34(22)	5.80	0.016	(4.58	0.03)
**Carrion**	48(45)	33(33)	61(61)	23.7	<0.001	(13.9	<0.001)
**Birds**	13(13)	8(7)	17(20)	3.84	0.050	(7.33	0.007)
**Amphibians**	7(7)	14(14)	0(0)	17.4	<0.001	(12.5	<0.001)
**Fish**	4(4)	4(3)	4(6)	n.s		n.s	
**Invertebrates**	29(32)	54(52)	5(7)	65.1	<0.001	(44.2	<0.001)
**Anthropogenic** **plants**	56(56)	51(52)	59(63)	2.81	0.093	(2.33	0.127)
**Natural plants**	27(28)	39(38)	14(14)	18.66	<0.001	(13.3	<0.001)
**Garbage**	14(13)	7(8)	20(19)	7.1	0.008	(5.15	0.023)
**n**	**223(190)**	**108(102)**	**111(84)**				

In parentheses: uninfected individuals (raccoon dogs with symptoms of sarcoptic mange excluded). Total: uninfected and infected individuals.

**Table 2:** Frequency of occurrence (FO) and the number of different mammal taxa (n) found in the stomachs of raccoon dogs in Estonia in autumn and winter 2010–2012 (n = 223).

Taxon	FO(%)	n
**A. Small mammals**		
** Cricetidae**	**14.8**	**33**
** ** *Microtus* sp.	8.1	18
** ** *Arvicola amphibius*	0.9	2
** ** *Myodes glareolus*	5.7	13
** Muridae**	**4.4**	**10**
** ** *Apodemus agrarius*	2.6	6
** ** *Apodemus flavicollis*	1.3	3
** **Unidentified	0.4	1
** Soricomorpha**	**10.5**	**24**
** ** *Sorex araneus*	0.9	2
** ** *Neomys fodiens*	0.4	1
** **Unidentified Soricidae	6.6	15
** ** *Talpa europaea*	1.3	3
** **Unidentified	1.3	3
**B. Carrion**		
** Carnivora**	**17.5**	**39**
** ** *Nyctereutes procyonoides*	9.0	20
** ** *Canis familiaris*	0.9	2
** ** *Vulpes vulpes*	0.4	1
** **Unidentified Canidae	1.7	4
** ** *Felis catus*	0.9	2
** ** *Lynx lynx*	0.4	1
** **Unidentified Felidae	0.9	2
** ** *Mustela nivalis*	0.4	1
** ** *Neovison vison*	0.4	1
** **Unidentified Mustelidae	1.3	3
** **Unidentified	2.6	6
** Artiodactyla**	**34.5**	**77**
** ** *Sus scrofa*	18.4	41
** ** *Capreolus capreolus*	9.0	22
** **Unidentified	5.7	13
**C. Other remains (unidentified)**	**12.7**	**29**

### Seasonal Changes

**Figure 1:**
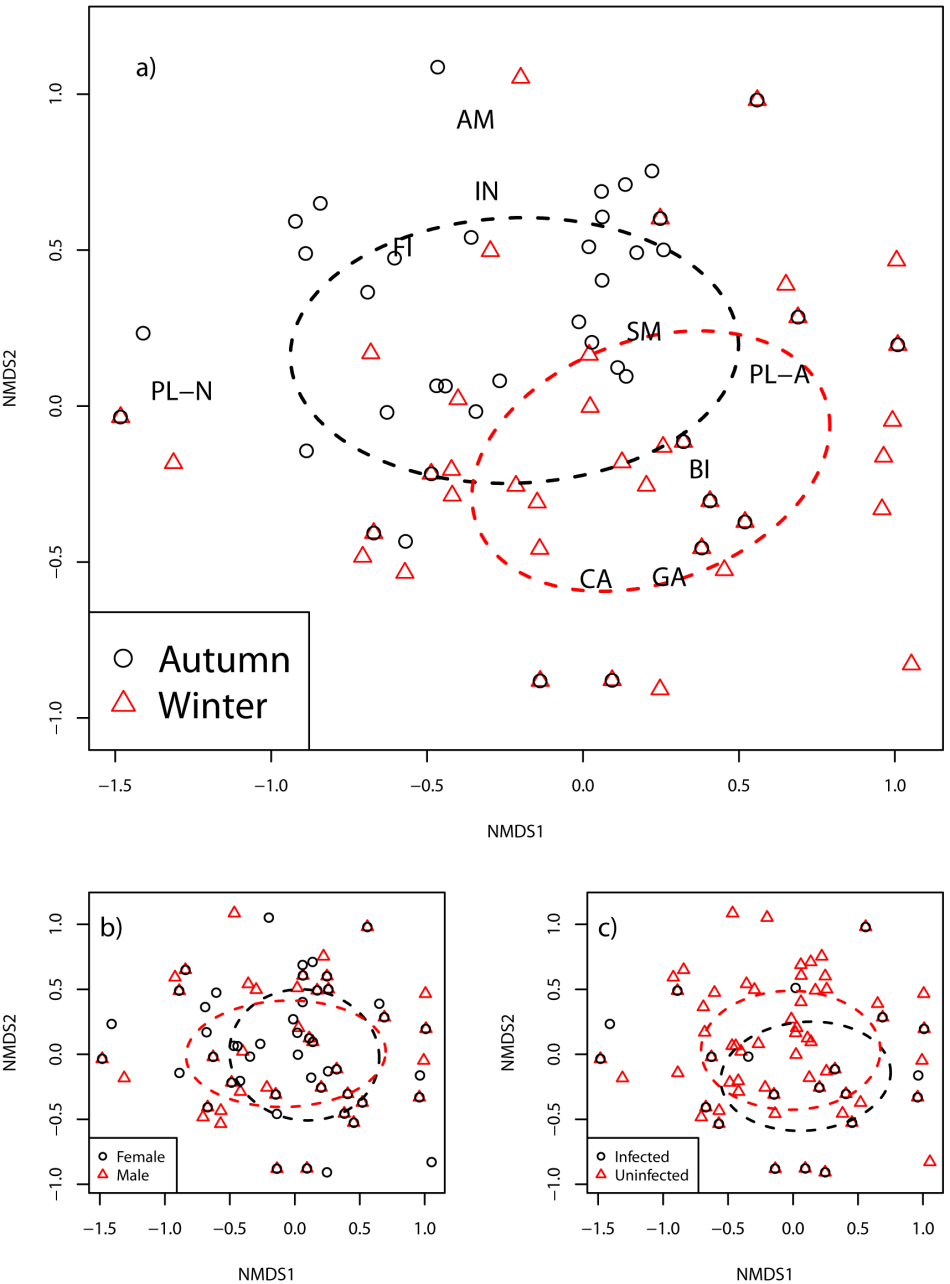
Non-metric multi-dimension scaling (NMDS; stress = 0.12) plots of raccoon dog diet between August and March in Estonia: a) samples are distinguished according to season (autumn or winter); b) the sex of the animal; or c) the mange infection status of the animal (infected or uninfected). Dashed ellipses indicate one standard deviation around the multivariate centroid of sample groups. PL-A – ‘anthropogenic plants’, PL-N – ‘natural plants’, BI – ‘birds’, SM – ‘small mammals’, CA – ‘carrion’, AM – ‘amphibians’, FI – ‘fish’, IN – ‘invertebrates’, GA – ‘garbage’.

Raccoon dog diet differed significantly between autumn and winter (PERMANOVA, pseudo-F = 28.2, R^2^ = 11.7, P = 0.001; Figure 1). The largest differences between seasons were in the consumption of ‘amphibians’ and ‘invertebrates’, which were consumed significantly more in autumn, whereas ‘small mammals’, ‘carrion’, ‘natural plants’ and ‘garbage’ were more frequent in the winter diet. ‘Anthropogenic plants’ was the only category that was consumed frequently in both autumn and winter (Table 1).

### Comparison of Males and Females

The only individual food category consumed significantly differently by female and male raccoon dogs was ‘birds’ (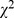 = 6.08, df = 1, p = 0.01; females: FO = 19.4%; males: FO = 8%) (Table S2). No significant difference was found in overall diet composition (PERMANOVA, pseudo-F = 1.86, R^2^ = 0.01, P = 0.15; Figure 1). The most frequently consumed food categories for both sexes were ‘anthropogenic plants’, which occurred in 56% of stomachs of both sexes, followed by carrion: FO = 52.4 for females and FO = 46.9 for males. Moreover, we did not find any significant difference in the volume of stomach food contents between male (n = 113; 84.2 ml ± 101.9 SD) and female raccoon dogs (n = 103; 108.7 ml ± 119.3 SD) (W = 12668, p = 0.27). Food niche breadth was very similar for the sexes, 2.79 for females and 2.65 for males, and food niche overlap between the sexes was very high (0.99).

### Evaluating the Impact of Sarcoptic Mange on Raccoon Dog Food Habits

Overall diet composition did not differ between raccoon dogs infected with sarcoptic mange (n = 33) and uninfected individuals (n = 190) (PERMANOVA, pseudo-F = 0.25, R^2^ = 0.001, P = 0.79; Figure 1). However, in comparison with uninfected animals, individuals with symptoms of sarcoptic mange consumed ‘carrion’ significantly more frequently during the whole study period (FO = 45.3 vs FO = 66.7; 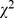 = 5.61, df = 1, p = 0.023), while healthy animals consumed significantly more invertebrates (FO = 31.6 vs FO = 12.1; 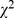 = 5.20, df = 1, p = 0.023) (Table 3); note that invertebrates were almost exclusively consumed in autumn (data not shown). No significant differences were found in the consumption of other food categories.

**Table 3:** Frequency of occurrence (FO) of different food items in the stomachs of raccoon dogs uninfected and infected with sarcoptic mange.

Food category	Uninfected	Infected	Uninfected/Infected	
	FO%		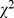	p-value
**Small mammals**	28.9	33.3	0.26	0.610
**Carrion**	45.3	66.7	**5.16**	**0.023**
**Birds**	12.6	15.2	n.s	
**Amphibians**	7.4	3.0	n.s	
**Fish**	4.2	3.0	n.s	
**Invertebrates**	31.6	12.1	**5.20**	**0.023**
**Anthropogenic plants**	56.3	54.5	0.04	0.85
**Natural plants**	28.4	21.2	0.74	0.391
**Garbage**	12.6	21.2	n.s	

When infected individuals were excluded from the seasonal comparison (autumn versus winter) the consumption of ‘birds’ became significantly different, being higher in winter (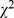 = 7.33, p = 0.007; Table 1). Exclusion of infected animals had no significant effect on other food categories.

### Co-occurrence of Food Items in Raccoon Dog Diet

‘Natural plants’ and ‘anthropogenic plants’ never co-occurred in the same stomach sample (Table 4). However, ‘anthropogenic plants’ were found alongside ‘small mammals’ significantly more often than expected by chance. ‘Anthropogenic plants’ also commonly co-occurred with ‘carrion’, though this relationship did not deviate significantly from random. Overall, individual raccoon dog stomachs tended to contain either broadly anthropogenic (‘anthropogenic plants, ‘garbage’ and ‘carrion’ commonly co-occurred) or broadly natural (‘invertebrates’, ‘amphibians’ and ‘natural plants’ co-occured) items, but mixtures of these categories were less frequent than expected (Table 4; Table S3; Figure S3). To some extent this reflected the availability of different food items in the different seasons, but the distinction remained largely intact within each season (Table S4).

**Table 4:** Co-occurrence of food items in raccoon dog diet.

	PL-A	PL-N	BI	SM	CA	AM	FI	IN	GA
**PL-A(125)**		**12.39**	−1.51	−**2.38**	−0.88	−1.21	0.44	−0.83	−1.17
**PL-N(61)**	0		0.99	1.91	1.32	−0.84	−1.43	−**2.1**	**2.09**
**BI(29)**	19	5		−1.36	−0.49	−0.3	1.08	0.15	−**3.36**
**SM(66)**	43	11	11		−1.32	−**1.78**	−0.51	−0.55	0.14
**CA(108)**	60	23	14	34		**2.59**	1.42	**3.58**	−**3.06**
**AM(15)**	10	5	2	7	2		0.75	−**5.79**	**1.55**
**FI(9)**	4	4	0	3	2	0		−0.52	1.13
**IN(64)**	36	22	7	19	18	15	3		0.97
**GA(31)**	19	3	10	8	22	0	0	6	

Numbers of samples in which a particular food type was recorded is shown in parentheses. Cells below the diagonal show the number of samples in which food types co-occurred. Cells above the diagonal show the standardised C-score (values below zero indicate co-occurrence; values above zero indicate separation). C-scores that deviate significantly from a random null model are shown in bold typeface. PL-A – anthropogenic plants, PL-N – natural plants, BI - birds, SM – small mammals, CA - carrion, AM - amphibians, FI - fish, IN - invertebrates, GA – garbage.

### Differences in Food Habits between Raccoon Dog and Red Fox

Overall autumn and winter diet composition differed significantly between raccoon dogs and red foxes (PERMANOVA, pseudo-F = 61.5, R^2^ = 0.17, P = 0.001), and food niche overlap between the two species was 0.86. When food categories were analysed separately, consumption of ‘small mammals’, ´carrioń, ‘invertebrates’ and ‘plants’ differed significantly between raccoon dogs and red foxes (Table 5). The most frequently consumed food categories for red foxes were mammals (‘small mammals’ and ‘carrion’), whereas for raccoon dogs it was ‘plants’.

**Table 5:** Frequency of occurrence (FO) of different food items in raccoon dog and red fox stomachs in autumn and winter.

Food category	Raccoon dog	Red fox		
	FO%		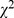	p-value
**Small mammals**	28.6	53.4	13.98	**<0.001**
**Carrion**	48.5	64.8	6.53	**0.01**
**Birds**	13.6	17.0	1.02	0.31
**Amphibians**	6.3	-	-	-
**Fish**	4.3	5.7	n.s	
**Invertebrates**	25.2	2.3	21.70	**<0.001**
**Plants**	82.5	44.3	48.90	**<0.001**
**Garbage**	14.1	11.4	1.69	0.688

## Discussion

### Temporal Variation in Raccoon Dog Diet: The Increasing Importance of Anthropogenic Plants and Carrion

Based on the results of this study, raccoon dogs in Estonia exhibit an opportunistic feeding strategy, and the consumption of different food items depends largely on their phenology. Carrion and anthropogenic plants appear to be the most important food sources for raccoon dogs in both autumn and winter, irrespective of sex. In comparison with earlier studies from Estonia there are marked differences in the consumption of small rodents and carrion (Figure S2; Information S1). Ungulate numbers and the hunting bag have increased during recent decades, which presumably explain the observed trends. Frequent consumption of ungulates can probably be also attributed to the high mortality of ungulates during the harsh winters in 2010–2012 (The Estonian Environment Information Centre). This interpretation is supported by the strong decline observed in the hunting bag size and winter track indices of both roe deer and wild boar in these winters (http://www.keskkonnainfo.ee). Ungulate carcasses are primarily left by large carnivores - ungulates are preferred prey of grey wolf (*Canis lupus*) and Eurasian lynx (*Lynx lynx*) in Estonia [30], [31] - but also by hunters. Previous studies from Lithuania [32], [33], Belarus [34], Poland [35], Russia [36] and Finland [37] have also shown that during their short active periods in winter, raccoon dogs mainly rely on ungulate carcasses. Carrion derived from dead carnivores was also found to form a significant food source for raccoon dogs in our study (17.5%). Unlike other scavengers, raccoon dogs do not avoid carnivore carcasses as a food source [38]. Moreover, hairs of raccoon dogs were found in 9% of stomachs, indicating that they consume the carcasses of conspecifics. Indeed, raccoon dog carcasses have long been used in Estonia as trap-line baits to lure other raccoon dogs.

The number of supplementary feeding sites for wild boar has increased in Estonia from approximately 2000 to 4000 since the early 1990s. Such sites are regularly provisioned with cereals, vegetables and fruit and sometimes also with the remains of hunted animals. The effect of this game management practice is clearly seen in the consumption of anthropogenic plants and perhaps also of carrion by raccoon dogs. While the plants consumed by raccoon dogs in the 1960s mainly consisted of the fruits and vegetative parts of naturally occurring plants [5], [39], both our study and that of Rätsepp [16] revealed that anthropogenic plants have come to represent the bulk of raccoon dog diet in Estonia during the past decade (FO>50%). A significant contribution of anthropogenic plants in the autumn and winter diet of raccoon dogs has also been demonstrated in Finland (FO = 55%) [37] and Germany (FO = 22.4%) [4]. Though natural food is available in autumn, the anthropogenic plants at supplementary feeding sites almost certainly represent a more concentrated and energy-rich food source.

### The Raccoon Dog as a Predator of Native Fauna

It has been suggested that the introduced raccoon dog may pose a threat to populations of ground nesting birds and amphibians, especially during their respective breeding seasons [5], [6], [7], [8]. However, the results of Naaber [5], Rätsepp [16] and this study suggest that the quantity of amphibians and birds in raccoon dog diet in Estonia depends on the season and that outside their respective breeding seasons, consumption of birds and amphibians generally remains low (though amphibians were a fairly important food source in autumn: FO = 13.9%). As most of the bird remains in this study belonged to passerines, raccoon dogs probably fed on carcasses rather than hunting the birds themselves. At a biogeographical scale, the consumption of birds by raccoon dogs has been shown to increase with latitude [4], and a general increase in carnivory with latitude has been reported for other omnivorous mammals [40].

A phenological effect is also evident in the consumption of invertebrates, which form an important part of raccoon dog diet in autumn (FO = 53.7%), but are rarely consumed in winter due to their low availability. In comparison with the 1960s [5], small mammals are consumed less frequently (46% vs 29.6%). The reduced importance of rodents and insectivores in raccoon dog diet found in this study probably reflects the increased availability of alternative supplementary food.

### Feeding Habits of Male and Female Raccoon Dogs

The food habits of female and male raccoon dogs were very similar. However, despite the fact that male and female pairs are strictly monogamous and usually forage together, the intake of birds was significantly higher in females, especially in early spring (March), when the difference was up to fivefold (data not shown). It is unclear why females might prefer birds more than males do, but there could be a nutritional benefit that is particularly important for female fecundity. Food niche breadth was also slightly broader for females, perhaps reflecting elevated energy demands in females prior to the mating season. The condition of female raccoon dogs is known to influence their litter size [41].

### Comparison of Dietary Habits with the Red Fox

Although dietary overlap between raccoon dogs and red foxes was significantly higher than in previous studies conducted in Europe [33], [35], raccoon dogs still exhibited more omnivorous and red fox more carnivorous feeding habits. Foxes consumed significantly more small mammals and carrion, whereas raccoon dogs ate more invertebrates and plants. Nevertheless, carrion and plants made up the bulk of both species’ diets in autumn and winter.

### Influence of Sarcoptic Mange on Raccoon Dog Food Habits

According to our study, overall raccoon dog diet did not vary significantly in relation to infection with sarcoptic mange. However, there were differences in the consumption of specific items. Individuals infected with sarcoptic mange consumed significantly fewer invertebrates (12.1% vs. 31.6%), but more carrion (66.7% vs 45.3%) in comparison with uninfected animals. This suggests that sarcoptic mange may influence foraging decisions or the ability to locate or catch live prey. It is probable that some of the carrion was consumed at or near to supplementary feeding sites, as carrion and anthropogenic plants tended to co-occur in stomachs more than expected by chance. As shown recently by Oja [42], infected raccoon dogs visit supplementary feeding sites more often than healthy individuals. The concentrated food sources available at feeding sites are probably more attractive to nutritionally impoverished infected animals than to healthy animals, which may prefer a diversity of food sources or choose not to visit the anthropogenically disturbed area as regularly.

The effect of sarcoptic mange was also apparent in the autumn-winter comparison, when uninfected individuals consumed significantly more birds in winter than in autumn. Carcasses of birds are likely to be more available during the winter (mortality due to the harsh conditions is higher in winter), and uninfected individuals are able to find them more easily; infected individuals are either less efficient foragers or concentrate more on supplementary feeding sites.

### Raccoon Dog Diet and Zoonotic Diseases

Since supplementary feeding sites attract rodents [42] they may facilitate the spread of another important zoonotic disease, alveolar echinococcosis, which is caused by the tapeworm *Echinococcus multilocularis.* In Estonia, about 30% of red foxes are infected with the parasite [15] and infected raccoon dogs have also been found recently (Laurimaa *et al*., unpublished data). We found that in over half of the cases where rodents appeared in stomach contents, cereals were also present, and the two items co-occurred in stomach samples significantly more often than expected by chance. Since rodents and anthropogenic food are also heavily consumed by foxes, feeding sites may promote inter- and intraspecific contacts and as a consequence increase the prevalence of the parasite. Similarly, frequent visits to supplementary feeding sites by animals with sarcoptic mange can increase contacts between infected and healthy individuals and therefore promote the spread of sarcoptic mange within and between species.

Considering that consumption of mammalian carcasses is the principle method for nematode transmission, and that *Trichinella* spp. are prevalent in raccoon dog populations in Estonia [43], the hunting practice of leaving the entrails and skins of quarry at supplementary feeding sites and in the forest could contribute to the spread of trichinellosis. Although rabies has been eradicated from Estonia following a successful anti-rabies vaccination campaign initiated in 2005, the disease remains at the border with Russia, and presents a genuine risk of spill-over. Thus, by mitigating the energetic demands imposed by harsh winter conditions and by promoting intra- and interspecific interactions, supplementary feeding sites and hunted remains may facilitate the spread of rabies and other severe infectious zoonoses.

## Supporting Information

Figure S1 **Sampling locations and numbers of raccoon dogs from different hunting districts in Estonia (the two-letter combinations represent the two first letters of the corresponding hunting district, for their full names see Table S1).**
(DOCX)

Figure S2 **Comparison of raccoon dog autumn and winter diet between three different study periods in Estonia (‘plants’  =  ‘anthropogenic plants’ and ‘natural plants; ‘other animals’  =  ‘invertebrates’, ‘amphibians’, and ‘fish’).**
(DOCX)

Figure S3 **Food categories that significantly co-occurred, or were significantly separate (based on Table S3).**
(DOCX)

Table S1 **Data for the sampled raccoon dogs in Estonia (see also Figure S1).**
(XLS)

Table S2 **Frequency of occurrence (FO) of different food items in female and male raccoon dog stomachs.**
(DOCX)

Table S3 **Food categories that significantly co-occurred, or were significantly separate (summary based on** **Table 4** **).**
(DOCX)

Table S4 **Co-occurrence of food items in raccoon dog diet in autumn and winter.**
(DOCX)

Information S1 **Comparison with two earlier studies performed in Estonia.**
(DOCX)
