# Supplementary material for: An Invasive Vector of Zoonotic Disease Sustained by Anthropogenic Resources: The Raccoon Dog in Northern Europe
Source: PLoS One. 2014 May 22;9(5):e96358. doi: 10.1371/journal.pone.0096358 (PMC4031070; doi:10.1371/journal.pone.0096358)
Supplement: Figure S1 — Sampling locations and numbers of raccoon dogs from different hunting districts in Estonia (the two-letter combinations represent the two first letters of the corresponding hunting district, for their full names see Table S1). (DOCX) [file pone.0096358.s001.docx]

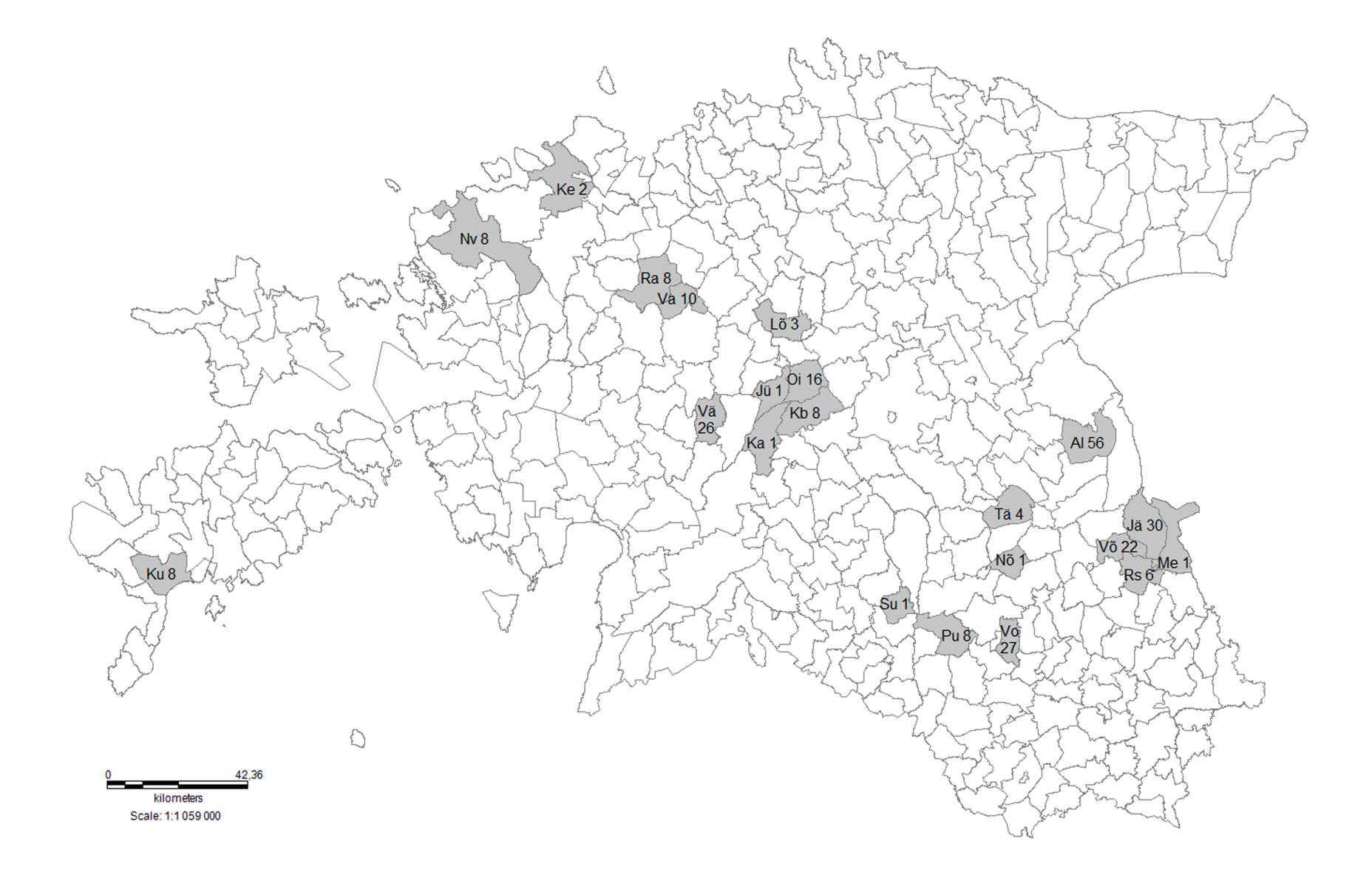


**Figure S1. Sampling locations and numbers of raccoon dogs from different hunting districts in Estonia** (the two-letter combinations represent the two first letters of the corresponding hunting district, for their full names see Table S1).
