# Supplementary material for: An Invasive Vector of Zoonotic Disease Sustained by Anthropogenic Resources: The Raccoon Dog in Northern Europe
Source: PLoS One. 2014 May 22;9(5):e96358. doi: 10.1371/journal.pone.0096358 (PMC4031070; doi:10.1371/journal.pone.0096358)
Supplement: Figure S2 — Comparison of raccoon dog autumn and winter diet between three different study periods in Estonia (‘plants’ = ‘anthropogenic plants’ and ‘natural plants; ‘other animals’ = ‘invertebrates’, ‘amphibians’, and ‘fish’). (DOCX) [file pone.0096358.s002.docx]

**Figure S2.** **Comparison of raccoon dog autumn and winter diet between three different study periods in Estonia** (‘plants’ = ‘anthropogenic plants’ and ‘natural plants; ‘other animals’ = ‘invertebrates’, ‘amphibians’, and ‘fish’).
