# Supplementary material for: An Invasive Vector of Zoonotic Disease Sustained by Anthropogenic Resources: The Raccoon Dog in Northern Europe
Source: PLoS One. 2014 May 22;9(5):e96358. doi: 10.1371/journal.pone.0096358 (PMC4031070; doi:10.1371/journal.pone.0096358)

**Figure S3.** **Food categories that significantly co-occurred, or were significantly separate (based on Table S3).** Natural food items (in green boxes) included ‘natural plants’ (PL-N in tables above), ‘invertebrates’ (IN), ‘amphibians’ (AM), ‘birds’ (BI) and ‘small mammals’ (SM); anthropogenic items (in red boxes) included ‘anthropogenic plants’ (PL-A), ‘carrion’ (CA) and ‘garbage’ (GA).


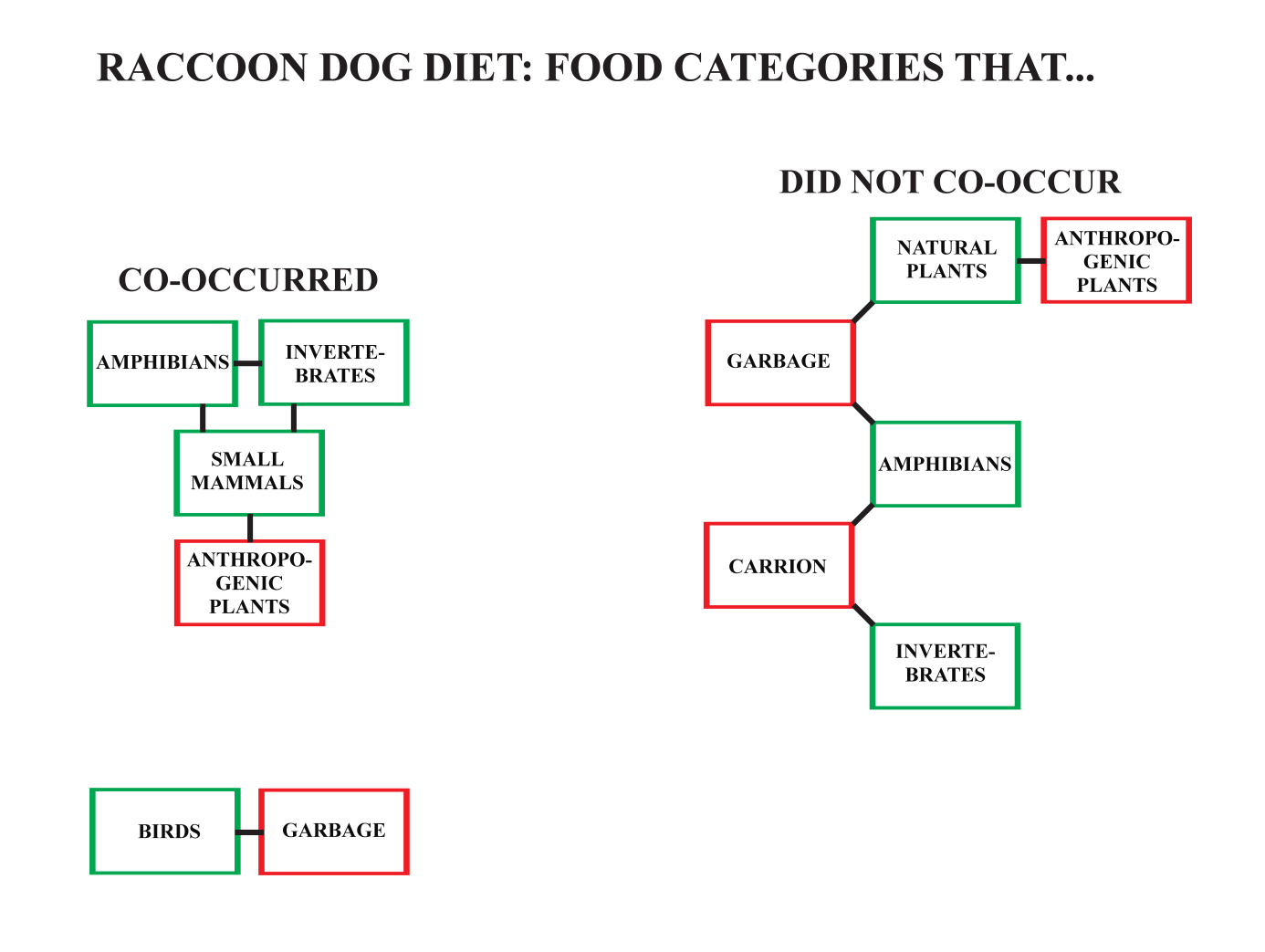

Supplement: Figure S3 — Food categories that significantly co-occurred, or were significantly separate (based on Table S3). (DOCX) [file pone.0096358.s003.docx]
