# Supplementary material for: An Invasive Vector of Zoonotic Disease Sustained by Anthropogenic Resources: The Raccoon Dog in Northern Europe
Source: PLoS One. 2014 May 22;9(5):e96358. doi: 10.1371/journal.pone.0096358 (PMC4031070; doi:10.1371/journal.pone.0096358)
Supplement: Table S2 — Frequency of occurrence (FO) of different food items in female and male raccoon dog stomachs. (DOCX) [file pone.0096358.s005.docx]

**Table S2.** **Frequency of occurrence (FO) of different food items in female and male raccoon dog stomachs.** Uninfected: individuals with symptoms of sarcoptic mange were excluded.

**Food category Total Uninfected Total Uninfected**

**FO% (female/male) χ^2^ p-value χ^2^ p-value**

**Small mammals** 32/27 32/25 0.79 0.375 1.16 0.282

**Carrion** 52/47 51/43 0.66 0.417 1.14 0.287

**Birds** 9/8 20/7 **6.08 0.014** **6.01 0.014**

**Amphibians**  6/8 7/8 0.38 0.537 0.13 0.715

**Invertebrates** 34/26 38/28 1.79 0.181 1.99 1.158

**Fish** 3/4 2/5 n. s n. s

**Anthropog. plants** 56/56 59/54 0.00 0.934 0.37 0.544

**Natural plants** 23/31 23/33 1.60 0.206 2.40 0.121

**Garbage** 16/13 14/13 0.22 0.636 0.07 0.796

**n 103/113 87/96**
