# Supplementary material for: An Invasive Vector of Zoonotic Disease Sustained by Anthropogenic Resources: The Raccoon Dog in Northern Europe
Source: PLoS One. 2014 May 22;9(5):e96358. doi: 10.1371/journal.pone.0096358 (PMC4031070; doi:10.1371/journal.pone.0096358)
Supplement: Table S3 — Food categories that significantly co-occurred, or were significantly separate (summary based on Table 4 ). (DOCX) [file pone.0096358.s006.docx]

**Table S3.** **Food categories that significantly co-occurred, or were significantly separate (based on Table 4).** Natural food items included ‘natural plants’ (PL-N), ‘invertebrates’ (IN), ‘amphibians’ (AM), ‘birds’ (BI) and ‘small mammal’s (SM); anthropogenic items included ‘anthropogenic plants’ (PL-A), ‘carrion’ (CA) and ‘garbage’ (GA). ^a^mixed indicates a mixture of the natural and anthropogenic categories.

| **Co-occurred** | | | **Did not co-occur** | | |
| --- | --- | --- | --- | --- | --- |
| **Natural** | **Anthropogenic** | **Mixed^a^** | **Natural** | **Anthropogenic** | **Mixed** |
| AM + SM | GA + CA | PL-A + SM |  |  | PL-N + PL-A |
| AM + IN |  | GA + BI |  |  | AM + CA |
| PL-N + IN |  |  |  |  | IN + CA |
|  |  |  |  |  | PL-N + GA |
|  |  |  |  |  | AM + GA |
