# Supplementary material for: An Invasive Vector of Zoonotic Disease Sustained by Anthropogenic Resources: The Raccoon Dog in Northern Europe
Source: PLoS One. 2014 May 22;9(5):e96358. doi: 10.1371/journal.pone.0096358 (PMC4031070; doi:10.1371/journal.pone.0096358)
Supplement: Table S4 — Co-occurrence of food items in raccoon dog diet in autumn and winter. (DOCX) [file pone.0096358.s007.docx]

**Table S4.** **Co-occurrence of food items in raccoon dog diet in autumn and winter.** Numbers of samples in which a particular food type was recorded is shown in parentheses. Cells below the diagonal show the number of samples in which food types co-occurred. Cells above the diagonal show the standardised C-score (values below zero indicate co-occurrence; values above zero indicate separation). C-scores that deviate significantly from a random null model are shown in bold typeface. PL-A – ‘anthropogenic plants’, PL-N – ‘natural plants’, BI – ‘birds’, SM – ‘small mammals’, CA – ‘carrion’, AM – ‘amphibians’, FI – ‘fish’, IN – ‘invertebrates’, GA – ‘garbage’.

**Autumn**

**PL-A PL-N BI SM CA AM FI IN GA**

**PL-A (55) 10.43** 0.14 **-2.01** -0.56 -1.66 -0.14 -1.83 -0.95

**PL-N (44)** 0 -0.46 1.66 1.01 0.29 -0.57 -0.11 **2.39**

**BI (9)** 4 4 -0.95 -0.8 -1 0.55 -1.07 -0.67

**SM (25)** 16 6 3 -0.51 0 -0.25 -1.96 0.56

**CA (38)** 19 12 4 9 **-2.53** 1.47 1.55 -1.08

**AM (15)** 10 5 2 7 2 0.8 **-3.78** 1.11

**FI (4)**  2 2 0 1 0 0 -0.11 0.56

**IN (58)** 32 22 6 17 15 15 2 -0.09

**GA (8)**  5 0 1 1 4 0 0 4

**Table S4 continued**

**Winter**

**PL-A PL-N BI SM CA FI IN GA**

**PL-A (69)**  6.12 -1.52 -1.27 -0.56 0.91 -0.34 -0.18

**PL-N (16)** 0 1.35 0.88 -0.79 -1.74 1.03 0.14

**BI (20)** 15 1 -0.64 1.12 1.03 -0.01 **-2.81**

**SM (40)** 27 4 8 -0.37 -0.28 0.01 0.37

**CA (70)** 41 11 10 25 1.42 0.48 -1.9

**FI (5)** 2 2 0 2 2 -1.66 1.14

**IN (6)**  4 0 1 2 3 1 -0.92

**GA (23)** 14 3 9 7 18 0 2
