# Supplementary material for: An Invasive Vector of Zoonotic Disease Sustained by Anthropogenic Resources: The Raccoon Dog in Northern Europe
Source: PLoS One. 2014 May 22;9(5):e96358. doi: 10.1371/journal.pone.0096358 (PMC4031070; doi:10.1371/journal.pone.0096358)
Supplement: Information S1 — Comparison with two earlier studies performed in Estonia. (DOCX) [file pone.0096358.s008.docx]

**Information S1. Comparison with two earlier studies performed in Estonia**

We also compared our data with the results of earlier diet studies performed in Estonia by Rätsepp [1] and Naaber [2]. Due to methodological considerations, statistical analysis could only be carried out with Rätsepp’s data. Data from Laanetu [3] were not used for temporal comparison due to the significantly shorter study period it employed.

Comparison of the results from this study with those from two earlier studies performed in Estonia revealed some significant trends (Figure S2). Consumption of ‘carrion’ and ‘other animals’ has gradually increased, whereas ‘birds’ has remained relatively stable. ‘Small mammals’ is the only food category which has decreased since the 1960s. Comparison between this study and the one conducted in 2003/05 revealed a considerable increase in plants ($\chi^{2}$=38.93, df=1, p<0.001), ‘carrion’ ($\chi^{2}$=14.26, df=1, p<0.001) and ‘other animals’ ($\chi^{2}$=7.01, df=1, p=0.01), while no significant changes were found in the consumption of ‘small mammals’ and ‘birds’ (p>0.05).

In the early 1960s small rodents were the main food of raccoon dogs (FO=45.5%) and carrion formed only a minor fraction (FO=11.9%) [5], but by the time of our study, ungulate remains had become the more frequently consumed item (FO=34.5%). Ungulate numbers and the hunting bag were low in Estonia during the 1960s and have continuously increased since then, which presumably explains their low importance in raccoon dog diet at that time. Even in comparison with the study by Rätsepp [1], which was conducted in 2003/05, the amount of carrion in raccoon dog diet has increased approximately twofold, from 23.5% to 48.4%. This can probably be attributed to the high mortality of ungulates during the harsh winters (low temperatures and deep snow cover) in 2010-2012 (The Estonian Environment Information Centre). This interpretation is supported by the strong decline observed in the hunting bag size and winter track indices of both roe deer and wild boar in these winters (<http://www.keskkonnainfo.ee>).

**References**

1. Naaber J (1971) Kährikkoer. Eesti Loodus 14: 449-455 (in estonian).
2. Laanetu N (1986) Ondatra - kiskja saakloom. Eesti Ulukid 4: 15-30 (in estonian).
3. Rätsepp M (2005) Kährikkoera (*Nyctereutes procyonoides*) ja punarebase (*Vulpes vulpes*) talvine toitumine Eestis. Bachelor Thesis, University of Tartu (in estonian).
